# Expression and clinical significance of CD31, CD34, and CD105 in pulmonary ground glass nodules with different vascular manifestations on CT

**DOI:** 10.3389/fonc.2022.956451

**Published:** 2022-09-15

**Authors:** Chen-ran Guo, Rui Han, Feng Xue, Lin Xu, Wan-gang Ren, Meng Li, Zhen Feng, Ben-chuang Hu, Zhong-min Peng

**Affiliations:** ^1^ Department of Thoracic Surgery, Shandong Provincial Hospital, Jinan, China; ^2^ Shandong University, Jinan, China; ^3^ Shandong First Medical University (Shandong Academy Of Medical Science), Jinan, China

**Keywords:** CD105, intratumoral micro-vascular density, ground glass nodules, vascular morphology, aniogenesis

## Abstract

Blood vessel passage on CT exerts a vital part in early diagnosis as well as treatment of carcinoma of the lungs. Intratumoral microvascular density (iMVD) has gradually become the focus of research on biological behavior, appearance, and evolution of malignant tumors nowadays. The aim of this paper was to verify whether there is a correlation between the iMVD and the vascular morphology of ground glass nodules (GGNs). A total of 109 patients with pulmonary GGN were classified into three groups (I,II, and III) according to the vascular morphology on CT, and their expression of CD31-, CD34-, and CD105-labeled iMVD was detected by the streptoavidin–biotin method, statistically analyzing the iMVD values of each group. The expression of CD31, CD34, and CD105 in different lung tissues was significantly different, with remarkably higher iMVD in lung cancer tissues than in adjacent normal lung tissues. In the imaging sort of types I, II, and III according to the means of vascular passage, the iMVD expression of CD31, CD34, and CD105 was significantly different between groups. These data suggest that the presence and the abnormal morphology of vessels seen within GGNs indicate the occurrence and progression of lung cancer in pathology. It offers a strong theoretical foundation for early diagnosis of carcinoma of the lungs, thus providing a more precise clinical diagnosis and prognosis of early-stage lung cancer.

## Introduction

According to “Global Cancer Statistics 2020” (1), there were approximately 2.2 million novel examples of carcinoma of the lungs and 1.8 million mortality worldwide in 2020, making lung cancer the second most common diagnosis and the most deadly malignancy in the world. Smoking, indoor and outdoor air pollution, and other related factors are inextricably linked to the continued high incidence of lung cancer (2–4), which imposes a heavy burden on our country’s health and economy. Despite the fact that comprehensive treatments such as surgery, chemoradiotherapy, and immunotherapy have improved significantly in recent decades, the whole 5-year survival rate for carcinoma of the lungs patients is still just 10%–20% in most countries among those diagnosed during 2010 through 2014 (1), due to the fact that most sick persons are in the late stages of diagnosis. Thus, early diagnosis of carcinoma of the lungs has vital practical significance.

In recent years, as low-dose multi-detector spiral CT has become a higher priority in lung cancer screening (5), more and more lung nodules are being checked. Through CT images, early carcinoma of the lungs mainly exists in the form of ground glass nodules (GGNs), which are divided into two categories: pure ground glass nodules (pGGNs) without solid components and mixed ground glass nodules (mGGNs) containing solid components.

Previous studies have found that the detection rate of pGGN in the population is 4.2% compared to 5.0% for mGGN (6), and the malignant rate of mGGN is higher than that of pGGN (7, 8). Therefore, the primary diagnosis of lung GGN by imaging features is the focus of the study. Among many imaging features of lung GGN, vascular penetration is an important feature. Gao (9) and Wang (10) found that the relationship between GGN and blood vessels was related to lung cancer, and abnormal blood vessels (including distortion, thickening, and branching) were more common in malignant GGN than normal blood vessels. Thus, it is worthwhile to investigate whether the relation under CT could be used to accelerate diagnosis of early lung cancer.

Many previous studies (11–13) have shown that angiogenesis is inextricably linked to tumor growth, metastasis, and disease prognosis. Folkman’s hypothesis that tumor growth depends on angiogenesis is supported by biological, pharmacological, and genetic evidence (14). Tumors larger than 2 mm in diameter require supplying vessels, including both pre-existing and new vascular ones (15).

Therefore, understanding the formation of tumor neovascularization provides more accurate help for studying the biological behavior, clinical treatment, and prognosis of malignant tumors. Immunohistochemical staining is often used to assess the number of new blood vessels in tumors. The measurement of microvascular density (MVD) by endothelial markers proposed by Weidner (16) is the mainstream method for semi-quantitative evaluation of the number of new vessels at present. Among them, CD31, CD34, and CD105 and other neovascular endothelial markers are commonly used markers. It has been shown that anti-endothelial antibodies such as anti-CD31 and anti-D34 are commonly used to assess angiogenesis, but these endothelial antibodies respond not only with emerging vessels, but also with normal vessels in tumor tissue (17). In contrast, CD105 is a 180-kDa homodimer membrane glycoprotein that binds transforming growth factor-β1 (TGF-β1) and transforming growth factor-β3 (TGF-β3), and the anti-CD105 antibody has a stronger affinity for proliferating vascular endothelial cells (ECs) (18). Therefore, CD105 is more conducive to evaluating the number of new blood vessels in tumor tissues. However, few studies have shown a correlation between blood vessels passing through GGNs and angiogenesis in lung cancer tissues. In the paper, we explored the expression condition of tumor neovascular EC markers CD31, CD34, and CD105 in GGNs by the IHC method, analyzing the difference of MVD in GGNs with different vascular manifestations under CT imaging. Then, statistical analysis of iMVD and other clinical data and clinicopathological features was conducted to verify the correlation between MVD and vascular morphology and location, and to provide new ideas and theoretical basis for the early imaging diagnosis, clinical treatment, and prognosis of non-small cell lung cancer (NSCLC).

## Materials and methods

### Patients

The samples in this study were selected from 109 patients who underwent radical lung cancer resection, whose systematic lymph nodes were sampled or dissected from November 2020 to March 2022 in the Department of Thoracic Surgery, Shandong Provincial Hospital. Forty subjects were male and 69 subjects were female, with an average age of 54.98 ± 10.13 years (range, 29–76 years).

The selected patients met the following criteria: (1) surgical resection was performed within 2 weeks after CT imaging and pathology was primary NSCLC; (2) the maximum diameter of GGNs was ≤2 cm; (3) there was no neoadjuvant therapy such as radiotherapy, chemotherapy, and immunotherapy before surgery; and (4) there was no synchronous tumor in other organs. Enrolled patients were staged according to the 8th Edition of the TNM Classification for Lung Cancer (Union for International Cancer Control/American Joint Committee on Cancer) (19), and then, the postoperative pathology of patients was classified as atypical hyperplasia (AAH), carcinoma *in situ* (AIS), microinvasive adenocarcinoma (MIA), and invasive adenocarcinoma (IAC) according to the WHO Classification of Thoracic Tumors (5th edition) (20). We obtained approval for the use of patients’ tumor tissues and clinical records as well as written informed consent. The study was in line with the principles of the Declaration of Helsinki and was revised and authorized by the Medical Ethics Committee of Shandong Provincial Hospital.

### CT examination method

The patient was admitted to the hospital and underwent supine plain CT scan plus chest enhancement with Philips Healthcare, Best, Netherlands on full inspiration. The scan area begins at the tip of the lung and ends at the corner of the rib diaphragm. High-resolution CT (HRCT) images were obtained using the following parameters: the section width was 0.625 mm, reconstruction gap could be 0.625 mm, pitch could be 0.984, tube voltage and current could be 120 kV and 250 mA, respectively, and the field of view could be 400 mm. All images were taken with a high-resolution 52.8 cm, 2,048 × 1,560 pixel grayscale monitor according to a standard lung window (window width: 1,500 HU; window position: −500 HU) as well as mediastinal window (window width: 350 HU; window position: 50 HU) for detection. In the enhanced CT scan, 60 ml of the nonionic iodinated agent iopromide was added to anterior elbow vein with a flow speed of 2.0–3.5 ml/s with high-pressure electrokinetic syringe, and picture collection was began 20–25 s after the completion of the injection, including arterial and venous phases as well as delayed scans.

### Analysis of vascular morphology on CT imaging

According to the multidisciplinary classification of lung adenocarcinoma based on the International Association for the Study of Lung Cancer, the American Thoracic Society, and the European Respiratory Society (21), GGN is subdivided into two types: mGGN and pGGN. The percentage of solid component of mGGN was calculated as follows: GGN and solid component diameters were measured under the lung window (the longest diameter and the shortest diameter at the largest cross-section were measured and averaged) (22) and the proportion of solid component diameter to GGN diameter was used to obtain the consolidation tumor ratio (CTR) (23). Then, GGN was analyzed according to whether there was blood vessel penetration in GGN and the morphology of the blood vessel penetration. Normal blood vessels taper off from the hilum to the distal end. If the blood vessel thickens as it enters the GGN, or deviates from its original trajectory, or both, we call it a thickened, twisted blood vessel and consider it abnormal. If multiple vessels in GGNs merge, we call this a sign of vascular convergence, which indicates a large amount of internal blood flow and may be associated with the tumor microenvironment in a neovascularized state. This more complex vascular morphology is also classified as abnormal vessels. In summary, GGNs can be divided into three types according to the morphology of penetrating blood vessels: Type I refers to GGNs without vascular penetration or with only vessels passing by the side; in Type II GGN, the vessel penetration mode is normal, and the expected vessel penetration path and thickness do not show significant morphological changes; Type III GGN is a type of GGN with abnormal vascular penetration patterns, such as thickening, hardening, or distortion of blood vessels, and other more complex patterns, such as vascular aggregation.

**Figure 1:**
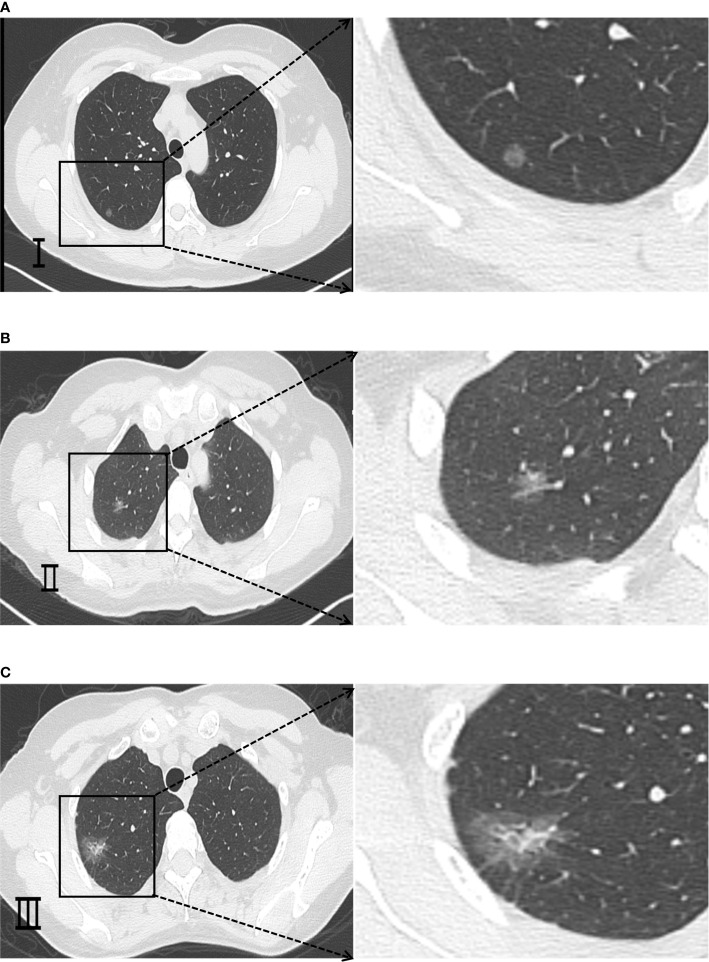
Representative CT imaging findings of GGNs classified according to different morphologies of passing vessels. **(A)** Type I is a GGN without vascular penetration or with only vessels passing by the side. **(B)** Type II is a GGN with normal pattern of vessel penetration and no obvious morphological changes in the expected path or thickness of the penetrating vessels. **(C)** Type III is a GGN with abnormal pattern of vessel penetration, such as thickened, stiffened or twisted vessels and other more complex patterns such as vascular clustering sign, etc.

The representative CT images of each type are shown in **Figure 1**. After all images were processed at the Department of Imaging of Shandong Provincial Hospital, the processed images were comprehensively analyzed and evaluated by two physicians having over 5 years of experience in diagnostic imaging of chest illness without knowledge of clinicopathological information about the patients. For each GGN subject, every radiologist independently showed the position of lesion, the category of GGN vessels, and the likelihood of malignancy. If there was any disagreement, a third senior physician was consulted.

### Histopathological classification

The pathological classification was based on the WHO Classification of Thoracic Tumors (5th edition) (20) and was divided into AAH, AIS, MIA, and IAC, while IAC was classified according to its subtype. The specimens were divided into groups according to the morphology of blood vessels, and pathological sections were made. The specimens were taken from the cancer tissue in the central part of the lesion. Meanwhile, paracancerous normal lung tissue was collected from normal tissue at least 2 cm from lesion center, and samples could be gathered carefully to make sure that paracancerous tissue was normal and not contaminated with tumor. All samples could be immobilized with 10% neutral formaldehyde solution, routinely implanted in paraffin, sectioned, and colored using hematoxylin–eosin (HE). Interpretation was performed unknowingly by two pathologists possessing over 5 years of experience in diagnostic pathology of thoracic tumors. If there is any disagreement, the third pathologist will join the discussion to help arrive at a conclusion.

### Immunohistochemical staining

We took sections (4 μm thick) of the lesions with more typical vascular penetration and normal lung tissue adjacent to the carcinoma, after gradient alcohol dehydration and xylene transparency, and immunohistochemical staining was performed using the streptoavidin–biotin method (s-p method) based on the information offered from the immunohistochemical kit. After dewaxing and rehydrating, the sections were processed by heating and cooling in citrate buffer (pH 6.0). Furthermore, they could be soaked using endogenous peroxidase blocker (3% hydrogen peroxide in methanol) for 30 min. The antigen was extracted with citric acid buffer (pH 6.0) and then the slices were incubated with 5% goat serum for 20 min, followed by IHC staining. Next, anti-CD31 monoclonal antibody (1:200, ZA-0568, Abcam, Cambridge, MA, USA), anti-CD34 monoclonal antibody (1:200, ZA-0550, Abcam, Cambridge, MA, USA), and anti-CD105 monoclonal antibody (1:300, EPR10145-12, Abcam, Cambridge, MA, USA) were applied as original antibodies in which the slices were cultured overnight at 4°C and washed. Sections were cultured for 1 h at room temperature using biotinylated goat anti-rabbit immunoglobulins (dilution 1:300; DAKOA/S) as secondary antibody. After restaining with hematoxylin, the slices could be sealed using neutral resin and visualized *via* the Olympus BX41 microscope (Ina-shi, Nagano, Japan)/Logene LG1000 (Wuxi, Jiangsu, China) Digital Camera System.

### Intratumoral microvascular density

Referring to the method proposed by Weidner (16) and Van Hoeff (24) to quantify the value of iMVD, two physicians having over 5 years of experience in the pathology of diagnosis performed the counts without knowledge of their CT images and pathological diagnosis. Both sides agreed on the criteria for reading microvessels before counting, and if the difference between the two counts is too great, a third pathologist will do the count alone and exclude results that differ significantly from the third person. An Olympus BX41 multi-head microscope was used to search the “hot spot” area with high microvascular density under a low-power field of 40 times, and then the number of CD31, CD34, and CD105 staining microvessels in this area was counted under a high-power field of 100 times. Five fields were randomly selected and the average value was taken after counting, which was the iMVD of the section. A single brown EC or a cluster of ECs is considered a microvessel as long as it can be clearly distinguished from tumor cells, other microvessels, and other connective tissue. Microvessels with thick walls or lumens larger than eight erythrocytes (24) and those in areas of sclerosis were not counted. The formation of a distinct lumen and the presence of erythrocytes in the lumen were not used as criteria for judgment.

### Statistical analysis

All results in this paper were studied using the SPSS v26.0 software program. All measures are expressed as`X ± S, and baseline data were statistically analyzed by one-sample *t*-test, nonparametric test, and ANOVA. In the between-group control, two groups were compared by one-sample *t*-test and multiple parts were contrasted using ANOVA. *p*-value <0.05 indicates statistical significance.

## Results

### Expression status of CD31, CD34, and CD105 in the tissues of carcinoma of the lungs as well as neighboring normal ones

**Figure 2:**
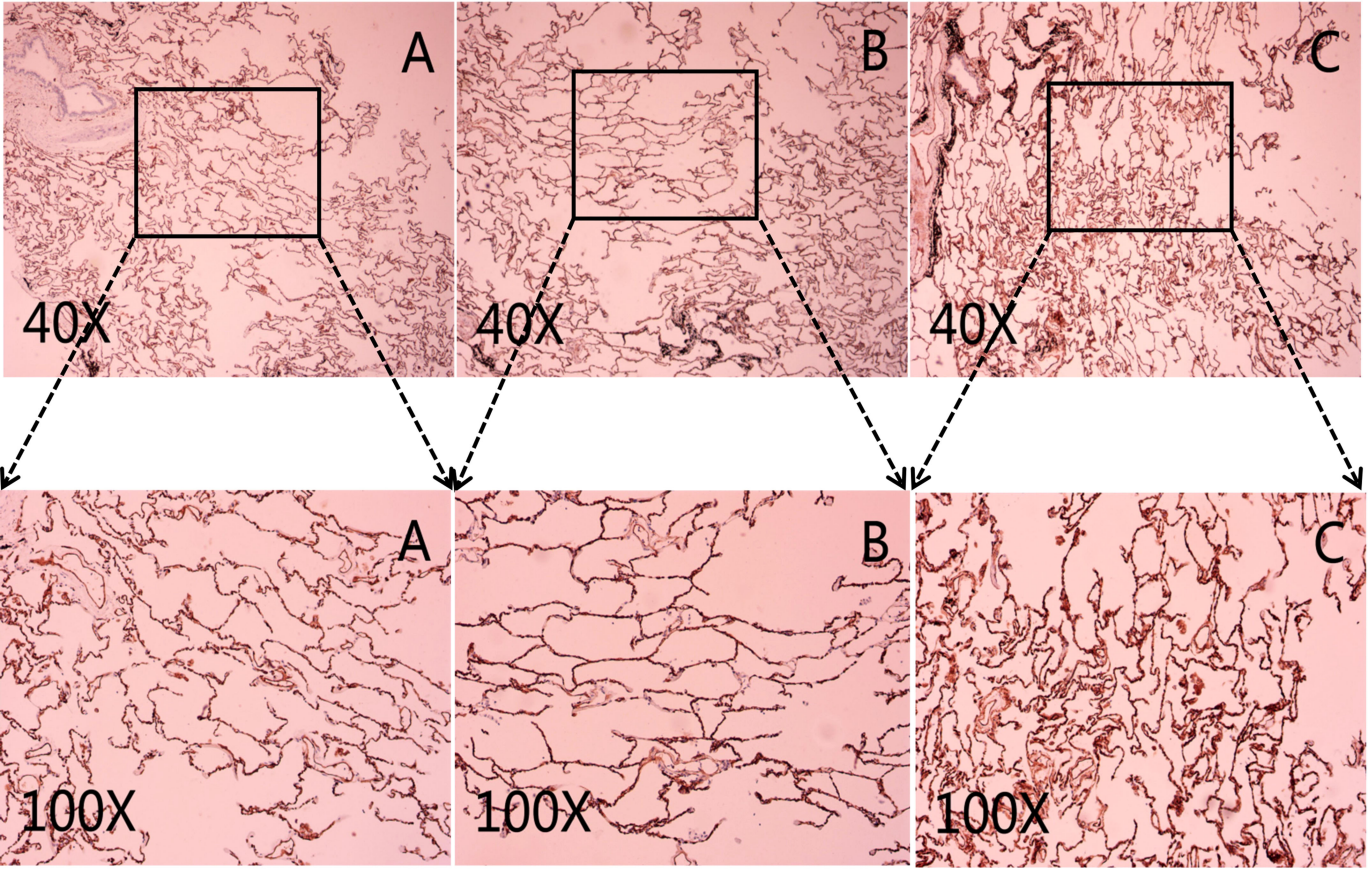
Representative micrograph in neighboring normal lung tissues. **(A)** Representative micrograph of CD31 expression in neighboring normal lung tissues. **(B)** Representative micrograph of CD34 expression in neighboring normal lung tissues. **(C)** Representative micrograph of CD105 expression in neighboring normal lung tissues. Black arrows point to the clearly stained neovascularization.

**Figure 3:**
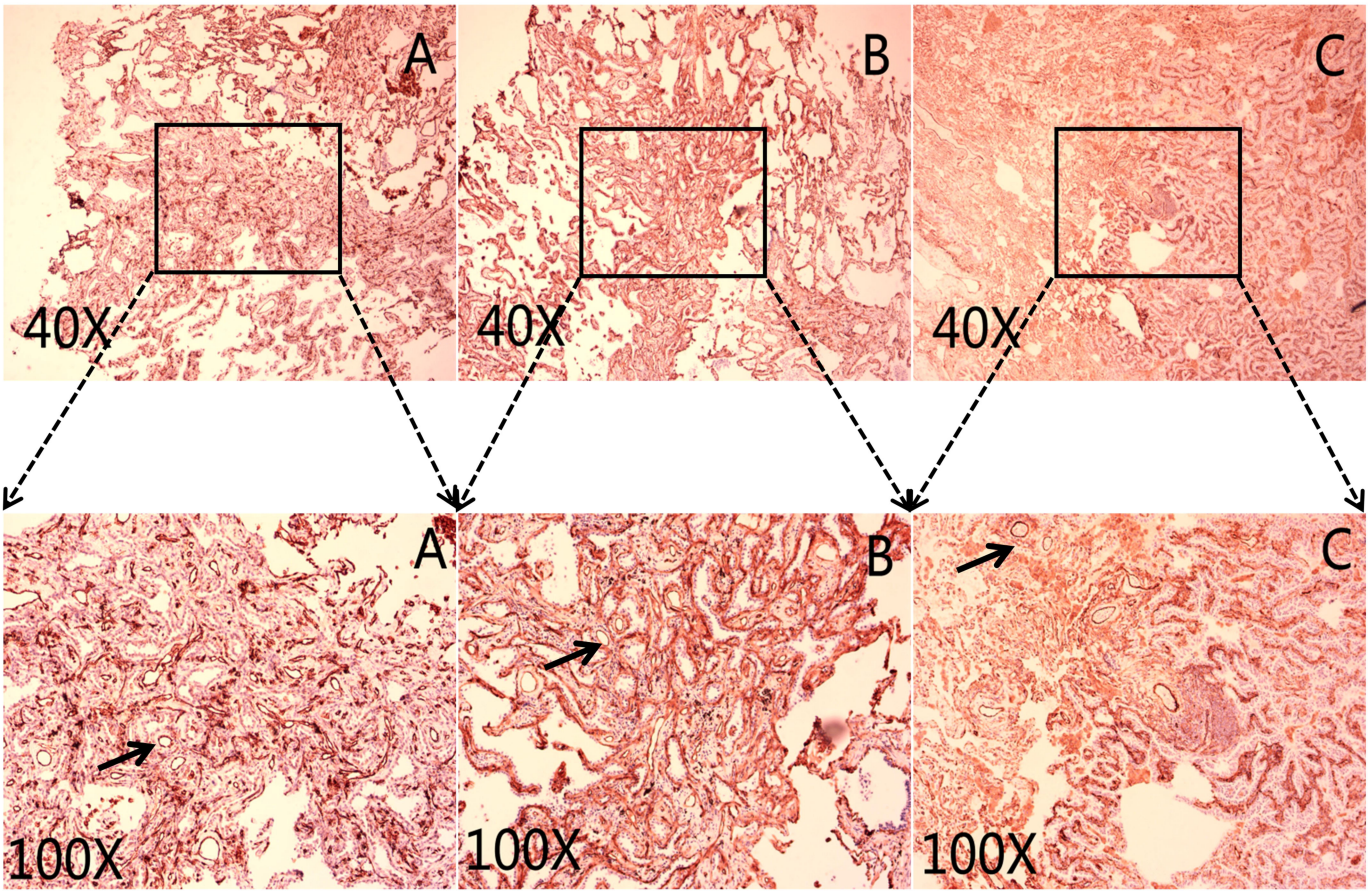
Representative micrograph in lung cancer tissues. The black arrows mark the microvascular endothelial cells. **(A)** Representative micrograph of CD31 expression in carcinoma of the lungs tissues. **(B)** Representative micrograph of CD34 expression in carcinoma of the lungs tissues. **(C)** Representative micrograph of CD105 expression in carcinoma of the lungs tissues. Black arrows point to the clearly stained neovascularization.

CD31 and CD34 had a widely positive expression in carcinoma of the lung tissues, “hot spot” areas were more numerous, and the positive expression was mostly located in the cytoplasm or cell membrane of ECs in microvessels and some larger luminal vessels, with brownish granules and clear boundaries with other surrounding tissues (**Figure 3**). In addition, there were positive expressions of CD31 and CD34 in neighboring normal lung tissues. CD105 could be expressed weaker in lung cancer tissues than CD31 and CD34, with a lighter brownish-yellow color and unclear boundary with the background. There were fewer “hot spots” in mature large vessel ECs, and their expression was negative (**Figure 2**). Most CD105-negative ECs expressed positive CD31 and CD34 markers (**Figure 3**). In addition, CD105 was rarely expressed in normal lung tissues neighboring tumors. Therefore, this study showed that the expression status of CD31, CD34, and CD105 was remarkably distinct in different tissues.

### Expression of IMVD tagged with CD31, CD34, and CD105 in carcinoma of the lung tissues, neighboring normal lung tissues, and precursor gland lesion tissues

**Figure 4:**
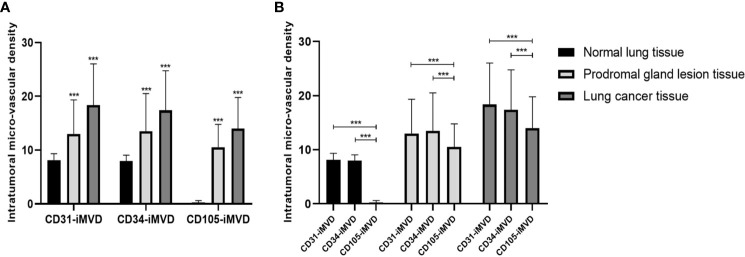
Interrelationships between the iMVD marked by immunohistochemical staining of CD31, CD34, and CD105 and GGN pathology types. **(A)** The values of CD31-iMVD are similar to CD34-iMVD, and their values are all higher than CD105-iMVD, suggesting that CD105-iMVD has a higher specificity than CD31-iMVD and CD34-iMVD. **(B)** With the increase of malignancy, CD31-iMVD, CD34-iMVD and CD105-iMVD have been significantly improved, indicating that they are sensitive indicators of the degree of tumor malignancy. ***indicates significance values p<0.001.

The expression status of CD31, CD34, and CD105 was closely related to tissue type, and their expression status was significantly different between lung cancer tissues, precursor glandular lesion tissues, and normal lung tissues adjacent to tumor (**Figure 4**). IMVD was significantly greater in lung cancer tissues than in normal lung tissues neighboring tumors (*p* < 0.01) (**Table 1**). There was no significant difference between CD31 and CD34 expression in normal tissues (*p*_CD31_ *_vs_*_CD34_ = 0.623 > 0.05), while a remarkable distinction was shown when comparing between CD31 and CD105, and between CD34 and CD105 (*p*_CD31_ *_vs_*_CD105_ = 0.000 < 0.05, *p*_CD34_ *_vs_*_CD105_ = 0.000 < 0.05). There was no evident distinction among CD31 and CD34 expression in anterior glandular lesion tissue (*p*_CD31_ *_vs_*_CD34_ = 0.243 > 0.05), while there was a significant difference when comparing between CD31 and CD105, and between CD34 and CD105 (*p*_CD31_ *_vs_*_CD105_ = 0.000 < 0.05, *p*_CD34_ *_vs_*_CD105_ = 0.000 < 0.05); otherwise, in lung cancer tissues, there were significant differences when comparing between CD31 and CD34, between CD31 and CD105, and between CD34 and CD105 (*p*_CD31_ *_vs_*_CD34_ = 0.019 < 0.05, *p*_CD31_ *_vs_*_CD105_ = 0.000 < 0.05, *p*_CD34_ *_vs_*_CD105_ = 0.000 < 0.05) (**Table 1**).

**Table 1:** y Expression of CD31-, CD34-, and CD105-labeled iMVD in lung cancer tissues, paracancer normal lung tissues, and proglandular lesions.

Pattern of tissue	*N* = 109	iMVD (mean ± SD)			*p-*value
		CD31-iMVD	CD34-iMVD	CD105-iMVD	
Normal lung tissue beside the carcinoma	15	8.13 ± 1.21	7.96 ± 1.09	0.23 ± 0.38	0.623_(CD31_ *_vs_*_CD34)_ 〈0.001_(CD31_ *_vs_*_CD105)_ 〈0.001_(CD34_ *_vs_*_CD105)_
Prodromal gland lesion tissue	32	13.01 ± 6.32	13.51 ± 7.00	10.53 ± 4.26	0.243_(CD31_ *_vs_*_CD34)_ 〈0.001_(CD31_ *_vs_*_CD105)_ 〈0.001_(CD34_ *_vs_*_CD105)_
Lung cancer tissue	77	18.37 ± 7.67	17.39 ± 7.36	14.01 ± 5.77	0.019_(CD31_ *_vs_*_CD34)_ 〈0.001_(CD31_ *_vs_*_CD105)_ 〈0.001_(CD34_ *_vs_*_CD105)_
	*p-*value	〈0.001	〈0.001	〈0.001	

In the WHO Classification of chest tumors (5th edition) (20) published by IARC in May 2021, lung adenocarcinoma in situ (AIS) and atypical adenomatoid hyperplasia (AAH) are classified as prodromal gland lesions; thus, this table lists them as separate tissue types.

### Correlation between IMVD of lung cancer tissues marked by CD31, CD34, and CD105 and clinicopathological parameters of patients

The expression of CD31-, CD34-, and CD105-labeled lung cancer tissue iMVD was significantly different among patients with a different smoking history (*p* < 0.05), tumor diameter (*p* < 0.05), and surgical procedure (*p* < 0.05). There were remarkable distinctions in the expression of CD34 and CD105 between patients with distinct pathological hypotypes of invasive adenocarcinoma (*p* < 0.05) and TNM periods (*p* < 0.05), and only CD105 was significantly different between patients with different percentages of solid tumor components (*p* = 0.009 < 0.05). There were no significant differences in age, gender, and body mass index (**Table 2**).

**Table 2:** The relationship between iMVD of lung cancer tissue marked by CD31, CD34, and CD105 and clinicopathological parameters of patients.

Clinicopathological parameters	*N* = 109	iMVD (mean ± SD)					
		CD31-iMVD	*p*-value	CD34-iMVD	*p*-value	CD105-iMVD	*p*-value
**Age**							
<60 years	71	16.91 ± 8.16	0.807	16.26 ± 7.73	0.794	13.02 ± 5.83	0.886
≥60 years	38	16.59 ± 6.78		16.24 ± 6.97		12.93 ± 5.15	
**Sex**							
Male	40	17.99 ± 7.64	0.083	17.60 ± 7.17	0.060	13.99 ± 5.48	0.103
Female	69	16.11 ± 7.67		15.48 ± 7.54		12.41 ± 5.59	
**Body mass index (BMI)**							
Normal	64	16.47 ± 7.21	0.427	15.75 ± 7.00	0.325	12.80 ± 5.22	0.907
Overweight	37	16.62 ± 8.19		16.09 ± 7.37		12.95 ± 5.55	
Fat	8	20.28 ± 9.05		21.05 ± 10.24		14.68 ± 8.57	
**History of smoking**							
Yes	42	24.70 ± 5.89	**0.000**	24.04 ± 5.50	**0.001**	18.41 ± 4.68	**0.000**
No	67	11.84 ± 3.20		11.37 ± 3.01		9.59 ± 2.62	
**Tumor diameter**							
≤1 cm	55	11.37 ± 2.95	**0.000**	11.24 ± 3.24	**0.000**	9.23 ± 2.44	**0.000**
>1 cm	54	22.32 ± 7.04		21.36 ± 7.04		16.82 ± 5.27	
**Consolidation tumor ratio (CTR)**							
≤50%	81	16.30 ± 7.64	0.196	15.44 ± 6.85	0.053	12.01 ± 4.75	**0.009**
>50%	28	18.22 ± 7.75		18.60 ± 8.66		15.83 ± 6.82	
**Pathological subtype of invasive adenocarcinoma**							
Lepidic	14	16.69 ± 6.71	0.098	14.21 ± 5.88	**0.007**	12.13 ± 4.29	**0.044**
Acinar+papillary	35	20.53 ± 7.37		20.15 ± 7.41		15.86 ± 6.16	
**Operation mode**							
Wedge resection	34	15.47 ± 7.63	**0.013**	15.72 ± 6.70	**0.006**	12.35 ± 5.39	**0.027**
Segmentectomy	53	15.85 ± 7.17		14.66 ± 7.06		12.22 ± 5.41	
Lobectomy	22	21.12 ± 7.73		20.93 ± 7.81		15.84 ± 5.59	
**TNM staging**							
0+I_A1_	77	15.58 ± 7.32	0.059	15.07 ± 7.06	**0.020**	12.07 ± 5.10	**0.030**
I_A1_+II	30	20.21 ± 7.84		19.69 ± 7.58		15.59 ± 6.12	

Bold indicates significance values p<0.05.

### Correlation between different vascular morphologies of GGNs and IMVD labeled with CD31, CD34, and CD105

The expression of CD31, CD34, and CD105 iMVD in different imaging subgroups was basically the same, and there were significant differences between the two groups. IMVD increases with the appearance of vascular permeability and the complexity of vascular morphology.

There was a remarkable distinction in the expression status of iMVD among types I, II, and III between two (*p*_I_ *_vs_*_II_ = 0.000 < 0.05, *p*_II_ *_vs_*_III_ = 0.000 < 0.05, *p*_I_ *_vs_*_III_ = 0.000<0.05), indicating that the tumor neovascularization labeled by this method may be related to the different morphology of tumor vessels. From type I, type II, to type III, the value of iMVD has a tendency to increase (**Table 3**).

**Table 3:** Correlation between GGN vascular imaging grouping and CD31-, CD34-, and CD105-labeled iMVD.

Image grouping	*N* = 109	iMVD (mean ± SD)					
		CD31-iMVD	*F/P* ^#^	CD34-iMVD	*F/P* ^※^	CD105-iMVD	*F/P* ^△^
TypeI	30	10.03 ± 2.24	*F*-value = 65.70 *p*-value < 0.000	9.28 ± 1.51	*F*-value = 76.47 *p*-value < 0.000	7.78 ± 1.50	*F*-value = 71.29 *p*-value < 0.000
TypeII	28	13.24 ± 3.16		12.99 ± 2.74		10.66 ± 1.89	
TypeIII	51	22.73 ± 6.97		22.15 ± 6.60		17.33 ± 5.04	

#p_(IvsII)_ < 0.001, p_(IIvsIII)_ < 0.001, p_(IvsIII)_ < 0.001; ^※^p_(IvsII)_ < 0.001, p_(IIvsIII)_ < 0.001, p_(IvsIII)_ < 0.001; ^△^p_(IvsII)_ < 0.001, p_(IIvsIII)_ < 0.001, p_(IvsIII)_ < 0.001。Type I is a GGN without vascular penetration or with only vessels passing by the side. Type II is a GGN with normal pattern of vessel penetration and no obvious morphological changes in the expected path or thickness of the penetrating vessels. Type III is a GGN with abnormal pattern of vessel penetration, such as thickened, stiffened, or twisted vessels and other more complex patterns such as vascular clustering sign, among others.

## Discussion

Nowadays, the widespread use of low-dose CT and the high incidence and mortality of lung cancer have increased the screening rate of GGNS (25, 26). GGN is an important imaging manifestation of early lung adenocarcinoma, and the increase of lung cancer incidence will be severe and inevitable in the future (27, 28). Thus, early diagnosis and therapy of lung tumor is crucial. AIS and AAH of the lung are classified as precursor glandular lesions in the WHO classification of thoracic tumors (5th edition) (20) published by the International Agency for Research on Cancer (IARC) in May 2021. Adenocarcinoma *in situ* can be removed from the lung adenocarcinoma classification in behalf of the good post-surgical prognosis at early-stage lung cancer. The 5-year living rate for early-period carcinoma of the lungs can be relatively high (even up to 100%), based on previous studies (29, 30). Therefore, early clear diagnosis of lung cancer patients is beneficial. In the paper, we used immunohistochemical s-p staining to verify the progressive increase of GGN malignancy, and the appearance and abnormal morphology of penetrating vessels, indicating the correlation between iMVD and GGN vascular penetration. Correlation provides advanced ideas and theoretical basis for the prognosis of NSCLC, and improves the effectiveness of identifying benign or cancerous GGN. Early-stage lung cancer often lacks typical symptoms; thus HRCT of the chest supports the clinical screening of it. GGN may present as lung adenocarcinoma, lung precursor adenopathy, benign lesions, or simple inflammation, and grow at an individual rate from small to large. Clinical studies (31, 32) suggest that the larger the size of the GGN, the greater the likelihood of malignancy. Therefore, in clinical practice, the treatment strategy for some small GGNs (especially subcentimeter pulmonary ground glass nodules) favors observation and follow-up over aggressive intervention. However, due to the lack of comprehensive diagnostic methods, also a small percentage of mGGNs with micro-papillary components may be a potential risk factor for poor prognosis. Clinicians often have difficulty making early diagnosis and determining the attributes of GGNs.

Consequently, it is crucial to grasp imaging features of smaller GGNs with vicious tendency, so that appropriate management can improve the survival rate of patients while releasing depression and improving their quality of life. In addition, Since most benign lesions do not require surgical treatment, unnecessary waste of medical resources is reduced.

Our previous study (33) showed that the CT characteristics of vicious GGNs like pleural traction sign, spiculation, and lobulation are helpful in the qualitative diagnosis of subcentimeter lung nodules, but many nodular lesions are small in size and not easily visible on CT. In contrast, clinicians can effectively make a qualitative diagnosis of small-sized GGNS with typical vessel penetration in small nodules. However, due to the lack of a strong pathological basis, vascular penetration is only a reference factor for the diagnosis of GGN. This study has found the theoretical basis for the relationship between the penetrating vessels and pathological diagnosis of GGNs, improving the status of vascular manifestations in GGN diagnosis.

Like normal tissue, tumors need blood vessels to supply blood for metabolism. Here, tumor angiogenesis forms a vascular supply network. Angiogenesis is a process in which the mechanisms of tumor cells mediate the migration and proliferation of vascular ECs (34). Hypoxia can increase the growth rate of tumor by affecting angiogenesis (35). Vascular endothelial growth factor A (VEGFA) secreted by hypoxic cancer cells initiates tumor angiogenesis by interacting with VEGF receptor 2 (VEGFR2) expressed by adjacent vascular ECs (36). Therefore, angiogenesis is closely associated with tumor growth. Many studies have shown that the number of tumor angiogenesis is closely related to tumor progression and patient prognosis. As an important clinicopathological parameter, iMVD can semi-quantitatively reflect the number of microvessels and angiogenesis in tumor, providing an important reference for the diagnosis and prognosis of patients with malignant tumor. Immunohistochemical staining is often used to assess and analyze the number of new vessels in tumors. The use of endothelial markers to measure microvascular density proposed by Weidner et al. (16) is currently the mainstream method for semi-quantitative assessment of angiogenesis, and neovascular endothelial markers such as CD31, CD34, and CD105 are currently the mainstream markers. CD34, a transmembrane protein encoded by the CD34 gene on chromosome 1q/32, is expressed through human hematopoietic cells from bone marrow and lymphatic system as well as ECs. It not only regulates the early incidence of blood cell differentiation but also functions as a cell–cell adhesion factor in ECs and hematopoietic progenitor cells (37). CD31 is a platelet endothelial cell adhesion molecule (PECAM-1) expressed by lymphatic vessels and vascular ECs, which can assist with evaluating angiogenesis and EC motility (38). Both are commonly used markers of vascular ECs and play a significant part in the detection of tumor angiogenesis. Current studies have determined that CD31 and CD34, being inexpensive and practical markers of vascular ECs, are significantly related to the evolution and prognosis of malignant tumors such as renal cell carcinoma (39) and melanoma (40). With the extensive research and clinical application of targeted anti-angiogenic drugs for tumors (41), research on anti-neovascular therapy for tumors is becoming a hot research area. In this study, their expression was shown to be highly expressed in cancer tissues, as well as significantly associated with smoking history, tumor diameter, and surgical procedure, which was similar to the findings of many previous studies. Subsequently, we observed a gradual increase in IMVD with the presence of vascular penetration and morphological abnormalities. Thus, the presence or absence of vascular penetrations and the abnormal morphology of the penetrating vessels are likely risk factors for increased malignancy of GGNs. Early diagnosis and characterization of GGNs with vascular penetration or abnormal morphology of the penetrating vessels often require more vigilance and caution. CD105, also known as endoglin, is a disulfide-linked homodimeric hypoxia-inducible cell membrane glycoprotein of 180 kDa, which acts as a co-receptor for TGF-β and is highly expressed in ECs with active tumor proliferation (42). A study has shown (43) that CD105 is involved in vascular development and remodeling as a suitable marker of tumor-associated angiogenesis and neovascularization, and its potential in diagnosis, prognosis, and therapy of several malignancies cannot be ignored. Miyata (44) et al. showed that CD105-MVD is a vital independent indicator of biochemical recurrence in sick persons after radical prostate cancer surgery. Moreover, it is also shown CD105 expression is related to the progression, stage, and patients’ prognosis of colorectal (45) and epithelial ovarian (46) cancers. Targeting CD105 has emerged as a new and powerful diagnostic and treatment strategy for malignancies (47). In the future, antibody-based CD105 targeting will play an irreplaceable role in the diagnosis and treatment of malignant tumors from the translation of preclinical research to clinical application of human cancer. In the paper, by verifying the expression of CD105 in different tissues and analyzing its correlation with different clinicopathological parameters of patients, CD105 was found to be more sensitive and specific as a marker of angiogenesis in early lung cancer. Meanwhile, CD105 can more specifically mark tumor angiogenesis without reacting with the original normal vascular ECs; thus, it is expected to be a new target for anti-tumor angiogenesis therapy in the future and a more ideal vascular EC marker compared with CD31 and CD34.

IMVD, as an indicator of the degree of angiogenesis, reflects the amount of current tumor angiogenesis and is considered to be an indicator of tumor progression and prognosis. At the same time, abnormal vascular penetration, as an imaging macro feature, is generally considered to be an important reference factor for the early diagnosis of GGN. Our study analyzed the correlation between angiogenesis and vascular penetration to find the theoretical basis for the relationship between the two. Furthermore, the relationship between pulmonary nodule vascular and pulmonary nodule pathologic malignancy was pointed out. It offers a firm theoretical foundation for early diagnosis of carcinoma of the lungs, and further provides more precise help for the clinical diagnosis and prognosis of early carcinoma of the lungs. The study showed that the two were statistically significant to a certain extent; that is, the presence or absence of vascular penetration and abnormal morphology of vascular penetration may be risk factors for increased GGN microvascular density.

There are also some limitations in this study. First of all, our experiment still proved the indirect relationship between abnormal vascular penetration and revascularization, and more basic experiments are needed to further verify the reasons for the relationship between the two. A reasonable guess might be that the microenvironment of neovascularization in the tumor has an effect on the adjacent large vessels, which, in turn, leads to the vascular imaging features. Although some studies (48) have suggested that the imaging features of vascular penetration described in this study are due to intratumoral desmoplastic reactive changes, it still lacks a strong underlying theoretical basis and therefore remains a focus and direction for future research. Secondly, because this study focuses on the diagnosis of early pulmonary small nodules and the operation time is a little closer to the present stage, there is a lack of postoperative survival follow-up data for patients with different iMVD and imaging characteristics. Therefore, the follow-up study on the prognosis of lung cancer patients with vascular permeability imaging characteristics is also worthy of attention.

## Conclusion

In this study, we confirmed that CD105 has higher specificity and sensitivity in reflecting the proliferation status of early-stage lung cancer vascular ECs, and is an ideal marker of lung cancer neovascularization. In addition, due to the high expression of neovascularization EC markers CD31, CD34, and CD105, the presence and abnormal morphology of penetrating vessels are pathological risk factors for the occurrence and development of lung cancer.

## Data availability statement

The original contributions presented in the study are included in the article/supplementary materials. Further inquiries can be directed to the corresponding author.

## Ethics statement

Written informed consent was obtained from the individual(s) for the publication of any potentially identifiable images or data included in this article.

## Author contributions

Z-MP and RH contributed to study design. C-RG and RH contributed to drafting of the article. C-RG, FX, and LX contributed to data collection. W-GR and RH contributed to pathological and immunohistochemistry evaluation. C-RG, ML and ZF contributed to Imaging analysis. Z-MP, B-CH and RH participated in data analysis and interpretation and led the article’s revision. All authors contributed to the article and approved the submitted version.

## Funding

This work was supported by Major Scientific and Technological Innovation Project of Shandong Province, PR China (2019JZZY021002).

## Conflict of interest

The authors declare that the research was conducted in the absence of any commercial or financial relationships that could be construed as a potential conflict of interest.

## Publisher’s note

All claims expressed in this article are solely those of the authors and do not necessarily represent those of their affiliated organizations, or those of the publisher, the editors and the reviewers. Any product that may be evaluated in this article, or claim that may be made by its manufacturer, is not guaranteed or endorsed by the publisher.

## Abbreviations

GGNs, ground-glass nodules; pGGN, pure ground-glass nodule; mGGN, mixed ground-glass nodule; NSCLC, non-small cell lung cancer; HRCT, high resolution computed tomography; HE, hematoxylin-eosin; s-p method, streptoavidin-biotin method; CTR, consolidation/tumor ratio; CT, computed tomography; LDCT, low dose computed tomography; ECs, endothelial cells; IHC staining, Immunohistochemical staining; iMVD, intratumoral microvascular density; TGF-β, transforming growth factor-β; AAH, atypical adenomatous hyperplasia; AIS, adenocarcinoma in situ; MIA, minimally invasive adenocarcinoma; IAC, invasive adenocarcinoma; IAR, International Agency for Research on Cancer; VEGFA, vascular endothelial growth factor A; VEGR2, Vascular Endothelial Growth Factor Receptor 2.
